# Relationship between nurses’ psychological capital and satisfaction of elderly cancer patients during the COVID-19 pandemic

**DOI:** 10.3389/fpsyg.2023.1121636

**Published:** 2023-01-27

**Authors:** Hui He, Na Zhu, Bei Lyu, Shengbao Zhai

**Affiliations:** ^1^School of Public Administration, Xiangtan University, Xiangtan, China; ^2^School of Economics and Management, Huaibei Normal University, Huaibei, China; ^3^Chinese Graduate School, Panyapiwat Institute of Management, Nonthaburi, Thailand

**Keywords:** satisfaction of elderly cancer patients, psychological capital, work engagement, job resources, job demands-resources model

## Abstract

**Introduction:**

As a special vulnerable group, the physical and mental health of elderly cancer patients has attracted much attention. However, few studies have focused on the impact of nurses’ mental state on the mental health of elderly cancer patients during the COVID-19 pandemic. In response to this literature gap, this study aims to explore the impact of nurses’ psychological capital on the satisfaction of elderly cancer patients. The job demands-resources model (JD-R) is used to further investigate how work engagement and job resources of nurses affect this relationship.

**Methods:**

The questionnaire survey was used to collect data, participants included 230 elderly cancer patients and their nurses from a tertiary first-class cancer hospital in China. Partial least squares structural equation modeling (PLS-SEM) was conducted with SmartPLS 3.3.9.

**Results:**

Nurses’ psychological capital has a significant positive impact on the satisfaction of elderly cancer patients during the COVID-19 pandemic. Nurses’ work engagement is an important mechanism for their psychological capital to affect the satisfaction of elderly cancer patients. In addition, nurses’ job resources positively moderate the relationship between their psychological capital and work engagement. The positive relationship between psychological capital and work engagement of nurses is stronger when they have abundant job resources.

**Discussion:**

These findings suggest that healthcare organizations should take the psychological capital of medical staff as an important means to improve their competitive advantage. It can improve the quality of medical services to obtain good performance by effectively developing and managing the psychological capital of medical staff. In addition, healthcare organizations should recognize the importance of providing adequate job resources for medical staff.

## 1. Introduction

Cancer is a chronic disease that seriously threatens human life. Patients not only need to face the suffering of disease, but also have to bear great mental pressure. About 50% of cancer cases occur in the elderly (Van Herck et al., 2021), and the elderly have a high incidence of most cancer types (DeSantis et al., 2019; Pilleron et al., 2019). The general public usually considers cancer as an incurable disease, which leads to the pain and despair of elderly cancer patients during cancer treatment. In addition, factors such as aging of physical function, economic pressure, the physical discomfort caused by cancer treatment, and changes in living habits after illness all contribute to the poor psychological health condition of elderly cancer patients. Research has found that anxiety, depression, and other negative emotions are particularly significant in elderly cancer patients, and 25% of elderly cancer patients may suffer from depression after diagnosis (Massie, 2004).

As a long-term, intense, and highly destructive crisis, the COVID-19 pandemic has caused social isolation and negative effects that seriously affect the mental health of people. In particular, elderly cancer patients are facing the triple threat of physical aging, tumor patients, and the spread of COVID-19. They have a stronger sense of anxiety compared to other groups. The risk of anxiety and depression in elderly cancer patients has increased by 50–60% since the COVID-19 pandemic (Li et al., 2021; Santomauro et al., 2021). Therefore, seeking effective measures to help elderly cancer patients adjust their mental state is not only a goal pursued by healthcare organizations but also an important research topic during the COVID-19 pandemic.

Patient satisfaction is a key indicator of the psychological state of cancer patients during the COVID-19 pandemic. It is helpful to understand the real thoughts of patients and has the function of predicting the potential needs and accurately grasping the changing trend of existing needs of patients. Patient satisfaction has been listed as one of the important indicators to promote national health by W.H.O. It also has been an important basis for healthcare organizations to improve service quality and allocate medical resources (Batbaatar et al., 2017). Patient satisfaction is affected by various subjective and objective factors (Cohen, 1996; Young et al., 2000; Schulmeister et al., 2005; Harrison et al., 2019). Most elderly cancer patients are hospitalized and have relatively close communication with medical staff (McLennon et al., 2013). Medical staff, especially nurses, are mainly responsible for daily life care (Fairbrother and Paice, 2005), psychological counseling, and professional medical care for cancer patients (Karlou et al., 2018). Therefore, as the direct provider of medical services with the closest contact with patients, service attitudes (Lake et al., 2016) and medical skills of nurses (Gebhardt et al., 2013) greatly affect patient satisfaction.

Moreover, the psychological condition of nurses is also an important factor affecting patient satisfaction. Especially during the COVID-19 pandemic, nurses caring for elderly cancer patients face heightened fear and uncertainty (Liu et al., 2021; Shen et al., 2021). They need to cope with the increased negative emotions such as panic and anxiety of patients caused by the COVID-19 pandemic (Joey DiCicco et al., 2019). At the same time, nurses must reasonably control their depression, anxiety, sadness, and other negative emotions (Robinson et al., 1991). In addition to ensuring the provision of normal medical services, nurses also need to prevent patients and their families from contracting COVID-19. They should take more thorough measures and undertake more complex work (Sullivan et al., 2022), which makes the workload of nurses increase sharply and bear great work pressure. Relevant research has shown that as a positive psychological state in the growth and development process of individuals, psychological capital is an important resource for nurses to effectively cope with work pressure and improve the quality of medical service supply (Hao et al., 2015). As a link connecting the internal health and external performance of nurses (Garrosa et al., 2010), the benign role of psychological capital plays a pivotal role in optimizing nursing quality and promoting patient satisfaction (Brunetto et al., 2016).

Nursing practice has shown that the work engagement of nurses plays an important role in patient satisfaction (Bacon and Mark, 2009; Ogbonnaya et al., 2018). Work engagement is an emotional and cognitive state associated with work, which has positive, substantial, and lasting characteristics. It manifests in employees’ efforts to participate in work and willingness to invest time and resources in their work (Schaufeli et al., 2006). The higher the enthusiasm and participation of nurses, the more energy, focus, and dedication they will devote to their work. It will help nurses to demonstrate better efficiency and performance, provide high-quality care, and effectively improve patient satisfaction. At the same time, the more satisfied patients are with care, nurses will feel that their efforts have been rewarded and develop a positive psychological expectation. This will make them to be more productive and devote themselves to their work (Schaufeli and Bakker, 2004), creating a virtuous circle. In addition, nurses are at high risk of burnout. Especially during the COVID-19 pandemic, nurses experience greater psychological stress and are more likely to suffer from burnout, which may reduce their work motivation and affect the quality of care services (Clarke, 2007). Job resources are the resources needed to achieve work goals, it includes career development opportunities, support from leaders and colleagues, team climate, participation in decision-making, and autonomy of work (Demerouti et al., 2001). These resources can contribute to the achievement of employees’ work objectives, and stimulate the growth, learning, and development of individuals. It is useful to reduce burnout and lead to higher engagement of employees (Schaufeli and Bakker, 2004). For nurses, different job resources will lead to different psychological and emotional effects on individuals, which will affect their work engagement. Generally speaking, obtaining rich job resources is conducive to positive psycho-emotional effects, and nurses will have high work enthusiasm and motivation (Bhatti et al., 2018).

Research has, respectively, confirmed psychological capital and work engagement of nurses have important effects on patient satisfaction, but few studies have combined nurses’ psychological capital and work engagement to explore their effects on elderly cancer patients’ satisfaction. Especially under the background of the COVID-19 pandemic, there is a lack of research on how the psychological state of nurses affects the satisfaction of elderly cancer patients and the impact mechanism needs to be further verified. According to the job demands-resources model (JD-R), psychological capital is a positive psychological state and an important individual resource of nurses. It can affect work engagement and other behaviors of nurses and then influence their work performance, such as patient satisfaction. In this process, social support, autonomy, and other job resources as external support elements may moderate the relationship between psychological capital and the work engagement of nurses.

**Figure 1:**
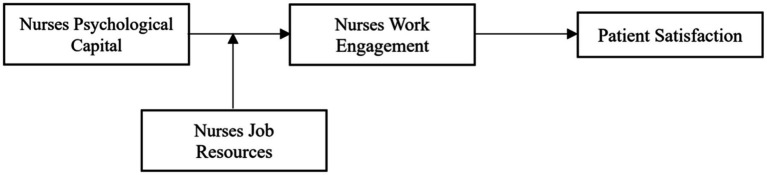
Theoretical model.

In summary, based on the JD-R model, this study explores the relationship between the psychological capital of nurses and the satisfaction of elderly cancer patients in the context of COVID-19 pandemic and examines whether nurses’ work engagement mediates this relationship. In addition, job resources are considered as a moderator to explore the boundary conditions of psychological capital and work engagement of nurses. The theoretical framework is presented in Figure 1. This study has the following contributions. Firstly, the antecedent research on the satisfaction of elderly cancer patients was improved and expanded from the psychological perspective of nurses. Secondly, the study combines psychological capital and work engagement to explain the influence mechanism of nurses’ psychological state on the satisfaction of elderly cancer patients, which enriches the current literature. Finally, according to the JD-R model, this study discusses the moderating role of nurses’ job resources in the relationship between their psychological capital and work engagement, it expands the literature on the boundary effect between psychological capital and work engagement. At the same time, this study provides a reference for healthcare organizations to consider how to take effective measures to optimize the management of medical staff, improve the quality of medical services and establish a good doctor-patient relationship.

## 2. Theoretical background and hypotheses

### 2.1. Nurses’ psychological capital and satisfaction of elderly cancer patients

The most consistent assessment of the effectiveness of cancer treatment interventions is the satisfaction of cancer patients with their care (Zimmermann et al., 2008), which is increasingly being included in cancer clinical trials (Lipscomb et al., 2007). Patient satisfaction is a subjective assessment that represents the cognitive and emotional responses caused by the interaction between patients’ expectations of ideal care and perceived actual care (Makhlouf et al., 2019). It is the most recognized and widely used medical services outcome item (Ong, 2000) and an important indicator to measure the quality of medical care (Donabedian, 1988). Measuring patient satisfaction also provides actionable insights for improving the healthcare system (Fontecha et al., 2019; Chung et al., 2020).

Cancer patient satisfaction is closely related to nursing care. Compared with other medical staff, nurses have the closest and longest contact with patients (Kim-Soon et al., 2017), and their nursing performance has an important influence on cancer patient satisfaction. Studies have shown that nurses’ performance and attitudes toward patients are related to their psychological capital. As a strategic resource, psychological capital has received increasing attention (Ardichvili, 2011). It includes self-efficacy, hope, resilience, and optimism (Newman et al., 2014; Luthans et al., 2015). Nurses’ psychological capital has a positive impact on the nursing performance of oncology nurses (Jung, 2019) and is conducive to improving the satisfaction of cancer patients. Specifically, when nurses have low psychological capital, they are more prone to empathy fatigue (Spence Laschinger and Nosko, 2015), work stress (Yin et al., 2018), and burnout (Gómez-Urquiza et al., 2017). These phenomena lead to negative states such as emotional exhaustion and decreased work enthusiasm (Aiken et al., 2002), which is not conducive to providing high-quality nursing services for patients. In contrast, high psychological capital is the motivation for nurses to work efficiently (Luthans et al., 2007), it significantly reduces burnout of nurses (Zhou et al., 2018) and promotes them to maintain a lasting work enthusiasm (Ren et al., 2021). Nurses with high psychological capital have stronger job embeddedness (Sun et al., 2012) and higher professional identity (Qiu et al., 2019). It can effectively improve nursing competence, achieve a high-quality nursing service supply, and ultimately improve patient satisfaction with medical services.

Most studies have examined the correlation between a single dimension of nurses’ psychological capital and patient satisfaction. For example, there is a positive correlation between the self-efficacy of nurses and patient satisfaction. Nurses need to have a high sense of self-efficacy to provide high-quality care for palliative care patients (Kurnia et al., 2018) and thus achieve high patient satisfaction (Aspinal et al., 2003). The emotional resilience of nurses is also positively correlated with patient satisfaction in hospice care, and patients cared for by high-resilient nurses tend to have higher satisfaction (Son and Park, 2021). In addition, optimistic nurses have a positive impact on patients. The optimism of nurses contributes to their work performance and increases patient satisfaction (Luthans et al., 2008). To comprehensively reflect the psychological state of nurses, this study selects four dimensions of self-efficacy, hope, resilience, and optimism to explore the relationship between the psychological capital of nurses and the satisfaction of cancer patients. Furthermore, there is a relative lack of research on elderly cancer patients in the cancer patient satisfaction literature. Elderly cancer patients have more intentions and opportunities to communicate with nurses, they are more sensitive to the emotions of nurses and more likely to be influenced by the nurses’ psychological capital. Accordingly, the following hypothesis is proposed.

*H1*: Nurses’ psychological capital is positively related to the satisfaction of elderly cancer patients.

### 2.2. The mediating role of nurses’ work engagement

Work engagement is a positive, fulfilling, and work-related psychological state, it consists of three aspects: dedication, focus, and vitality (Schaufeli and Bakker, 2004; Schaufeli et al., 2006). There are many factors affecting work engagement, including leadership style (Decuypere and Schaufeli, 2020), organizational climate (Rožman and Štrukelj, 2021), and diverse HR practices (Luu et al., 2019) at the organizational level. Psychological flexibility (Holmberg et al., 2020), proactive personality (Hu et al., 2021), self-efficacy, and resilience (Bakker and van Wingerden, 2021) at the individual level. According to the JD-R model, both personal and job resources can have a significant impact on work engagement (Bakker and Demerouti, 2017). Employees will generate work engagement if they have sufficient resources to handle job demands (Bakker and Demerouti, 2008). Psychological capital is a collection of positive psychological resources (Thompson et al., 2015), including hope, efficacy, resilience, and optimism (Luthans et al., 2015). Psychological capital has proved to be an effective personal resource (Newman et al., 2014) that can help employees maintain work engagement (Simons and Buitendach, 2013; Gupta et al., 2017).

Psychological capital has a positive impact on work attitudes, psychological state, and performance of employees (Peterson et al., 2011; Adil and Kamal, 2016). It is also a significant predictor of work engagement (Joo and Lee, 2017). Studies have found that employees with higher psychological capital are more likely to have positive emotions, which helps to increase their vitality, focus, and dedication at work, and thus generates higher work engagement (Gupta et al., 2017; Karatepe and Avci, 2017). In addition, psychological capital is considered a positive resource for coping with negative outcomes such as burnout and workplace violence (Sui et al., 2019). When facing difficult clinical problems, nurses with high psychological capital can immediately sense the positive personal resources they possess, and usually will not give up or be frustrated easily. They will actively think about solutions to problems and maintain the ambition to accomplish their goals (Yang et al., 2020), generating high work engagement. Relevant studies have also confirmed that nurses’ psychological capital is positively associated with their work engagement (Pan et al., 2017; Wang et al., 2017). Accordingly, the following hypothesis is proposed.

*H2*: Nurses’ psychological capital is positively related to their work engagement.

Patient satisfaction is a useful performance indicator for healthcare providers (Heidegger et al., 2006; Jha et al., 2008). In nursing practice, the work engagement of nurses has strategic importance. Because as the largest group of healthcare providers, the work engagement of nurses may affect the quality of care, work performance, patient satisfaction, and even patient safety (Keyko et al., 2016; Perreira et al., 2018). Work engagement is the prerequisite for positive work behaviors. Nurses with high work engagement have a strong sense of initiative that facilitates optimal nursing care (Yongxing et al., 2017; Lisbona et al., 2018). Highly engaged nurses can proactively respond to patient needs and improve patient satisfaction with care (Bacon and Mark, 2009). At the same time, the work engagement of nurses tends to be positively correlated with their job satisfaction, which is positively related to patient satisfaction (Mrayyan, 2006; Ferrara et al., 2013).

Dedicated nurses tend to have positive emotions and are more likely to have work-related pleasure, which will increase their occupational health (Gandhi et al., 2014; Khamisa et al., 2015). This state of well-being promotes successful performance (Ferrara et al., 2013) and positively affects patients’ perceptions of nursing quality (Papastavrou et al., 2014). Research has found that nurses with high work engagement are often energetic, they can respond effectively to job demands and have more efficient performance, which has a positive impact on patient satisfaction (Schaufeli et al., 2006). High levels of work engagement among nurses are associated with positive quality of care outcomes, including fewer medical errors (Tsiga et al., 2017), more professional cognition (Cao et al., 2020), and higher patient satisfaction (De Simone et al., 2018). Conversely, when nurses’ work engagement is low, they typically exhibit low morale, emotional exhaustion, and a lack of enthusiasm for their work. It will negatively affect patient experience and reduce the quality of care and patient satisfaction (Willard-Grace et al., 2021). Studies have explored the influencing factors of cancer patient satisfaction from the aspects of treatment outcome, hospital environment, medical equipment, nursing technology (Wiggers et al., 1990), and nursing accessibility (Sitzia and Wood, 1998). Few scholars have directly discussed the impact of nurses’ work engagement on patient satisfaction, and research on elderly cancer patients is rarer. Elderly cancer patients are a special patient group, their satisfaction studies have similarities with ordinary patients. Thus, this study proposes the hypothesis.

*H3*: Nurses’ work engagement is positively related to the satisfaction of elderly cancer patients.

According to the analysis of hypothesis 2, nurses with high psychological capital will have positive work effects and can positively influence their work engagement. Based on the analysis of hypothesis 3, nurses’ work engagement has an important influence on their performance. Nurses with higher work engagement can give full play to their work potential and initiative, which can improve the quality of care and patient satisfaction. In summary, the following hypothesis is proposed.

*H4*: Nurses’ work engagement plays a mediating role between psychological capital and the satisfaction of elderly cancer patients.

### 2.3. The moderating role of nurses’ job resources

Job resources refer to the physical, psychological, social, and organizational resources owned by employees in the work environment. These resources usually have functions of achieving work goals, reducing physical and psychological exertion associated with job demands, and motivating individual growth, learning, and development (Demerouti et al., 2001; Hobfoll, 2002). Common job resources include job control, performance feedback, support from leaders and colleagues, job autonomy, and career development opportunities (Schaufeli and Bakker, 2004; Bakker and Demerouti, 2007). The JD-R model suggests that job resources play an important role in initiating the motivational process. Individuals with sufficient job resources are more likely to be motivated to complete high-demand tasks, generate higher work engagement, and improve work performance (Bakker and Demerouti, 2007; Bakker and Demerouti, 2008). Job resources can stimulate individuals from both internal and external aspects to achieve positive outcomes. As an internal motivation factor, job resources can meet the basic needs of people and promote the growth and development of employees. As an external incentive factor, job resources can help employees to enhance their core self-concept and improve their well-being, which promotes their efforts to fulfill job demands and job roles and ultimately achieve job goals (Schaufeli and Bakker, 2004; Bakker and Demerouti, 2017).

According to the JD-R model, translating job demands into work engagement requires certain job and personal resources (Bakker and Demerouti, 2007). The work of nurses often includes stressful and difficult situations, it also has high emotional requirements. Therefore, to encourage high work engagement, nurses as boundary crossers in healthcare organizations need to be supported by more job and personal resources (Othman and Nasurdin, 2013). Nurses with high psychological capital tend to have more positive psychological states. When they have rich job resources at the same time, they are more likely to feel that work goals are highly consistent with their own. As a result, these nurses are intrinsically and extrinsically motivated to be more focused on their goals and drive themselves to generate greater work engagement (Xanthopoulou et al., 2009). When nurses are engaged in nursing services, they invest personal resources such as psychological capital. If they can get support from superiors and colleagues, and enjoy job autonomy, job control, and other job resources, the personal resources of nurses will be supplemented and their professional ability will be developed (Akkermans et al., 2013), which will generate stronger engagement. Conversely, when nurses invest inherent personal resources in their work but do not receive the appropriate job resources as supplements, they will reduce the supply of personal resources to prevent the threat of resource depletion due to deprivation of their resources (Ito and Brotheridge, 2003), thus decreasing their work engagement. In summary, the following hypothesis is proposed.

*H5*: Nurses’ job resources play a moderating role between their psychological capital and work engagement. Compared with low job resources, the positive relationship between psychological capital and work engagement is stronger under high job resources.

## 3. Materials and methods

### 3.1. Sample and data collection

In the present study, the questionnaire survey was conducted in a tertiary first-class hospital in central China, which mainly focuses on oncology treatment. The hospital integrates medical treatment, research, teaching, prevention, and rehabilitation, it is a research center for oncology prevention and treatment in central China and has received 1.26 million inpatients. Patients are admitted from all provinces in China, with a wide geographical distribution and a representative sample. The study consisted of two groups: elderly cancer patients and their care nurses. The inclusion criteria for elderly cancer patients are as follows: (1) over 60 years old; (2) cancer diagnosed between July 2020 and July 2022 by pathological examination and combined with clinical signs and symptoms; (3) able to communicate effectively; and (4) voluntarily accepting the questionnaire survey. Exclusion criteria are as follows: (1) patients with visual or hearing impairment and (2) patients with other serious comorbidities (severe heart disease, etc.) that prevented them from participating in this study. The inclusion criteria for nurses are as follows: (1) have a professional qualification of nurse and within the valid registration period; (2) continuous work in the current department for more than 6 months; and (3) voluntarily participating in this study. Exclusion criteria are as follows: (1) nurses in training, or not working due to sickness or maternity leave and (2) advanced training, internship, or temporary nurses.

Investigators were uniformly trained before the formal survey to ensure that their instructional language was standardized and explanations were clear. With the consent of the nursing departments of the survey hospitals, paper questionnaires were first distributed to several patients and nurses in May 2022 for pre-survey. Based on the feedback from the pre-survey, the questionnaire was revised to ensure that there was no ambiguity in context and sentences. A formal study was conducted in July 2022 in the form of a paper questionnaire. Following the principle of informed consent, elderly cancer patients and nurses who met the sample selection criteria were surveyed. The investigator explained the purpose, significance, and method of completion of the study before filling out the questionnaire. Due to the age and knowledge limitations of the elderly cancer patient, the investigator explained the content of the questionnaire in detail and then answered the questions raised by the respondents exactly and objectively before the survey. In this study, nurses recovered 279 questionnaires and patients recovered 293 questionnaires. Two hundred and sixty-two matching questionnaires were finally obtained after screening, and invalid questionnaires with many missing values were deleted. Two hundred and thirty valid questionnaires were retained in total, with a valid return rate of 87.79%.

### 3.2. Measurements

#### 3.2.1. Dependent variable

Satisfaction of elderly cancer patients was measured by the 5-item patient satisfaction scale developed by Tei-Tominaga and Sato (2016). Example items were *I am satisfied with the nursing care during my hospitalization. I am satisfied with this hospital overall* Items were rated on a 5-point Likert scale from 1 = *strongly disagree* to 5 = *strongly agree*. Cronbach’s alpha for this scale was 0.924.

#### 3.2.2. Independent variable

Nurses’ psychological capital was measured by the 8-item measurement scale (Megeirhi et al., 2018). For each dimension of the self-efficacy, hope, resilience, and optimism, the nurses were asked to assess their psychological capital using a 6-point Likert scale from 1 = *strongly disagree* to 6 = *strongly agree* (e.g., *I feel confident in representing my work area in meetings with management*.). Cronbach’s alpha for this scale was 0.914.

#### 3.2.3. Mediating variable

Nurses’ work engagement was measured by the 9-item work engagement scale (Schaufeli et al., 2006). The scale consisted of three dimensions: vitality, concentration, and dedication. Each dimension had three items. Nine items were rated on a 7-point scale from 1 = *never* to 7 = *always*. (e.g., *I feel myself bursting out of energy in my work. I am immersed in my work*.). Cronbach’s alpha for this scale was 0.939.

#### 3.2.4. Moderating variable

Three job resources were included in the questionnaire—autonomy, possibilities for professional development, and social support from colleagues. Autonomy was assessed with a three-item scale, based on the job content instrument of Karasek et al. (1998). Example items where *I can decide how I execute my work*. *I have the freedom to decide how I do my work* (1 = *never*, 5 = *always*). Possibilities for professional development were measured with the three-item scale of Bakker et al. (2003), including *My work offers me the opportunity to learn new things* and *I have sufficient possibilities to develop myself at work* (1 = *neve*r, 5 = *always*). Social support was measured with three items of the scale developed by Van Veldhoven and Meijman (1994). Example items are *Can you ask your colleagues for help if necessary?* and *Can you count on your colleagues when you face difficulties at work*? (1 = *never*, 5 = *always*). Cronbach’s alpha for this scale was 0.932.

#### 3.2.5. Control variables

Some demographics were controlled for in the study. Control variables of nurses included age (1 = 30 years or below, 2 = 31–35 years, 3 = 36–40 years, 4 = 41–45 years, 5 = 45+ years), education (1 = technical secondary school, 2 = junior college, 3 = bachelor’s degree, 4 = master’s degree or above), marital status (1 = married, 0 = unmarried), tenure (1 = 1–5 years, 2 = 6–10 years, 3 = 11–15 years, 4 = 16–20 years, 5 = 20+ years), hire type (1 = staff nurse, 2 = employment nurse, 3 = contract nurse), title (1 = nurse, 2 = nurse practitioner, 3 = nurse-in-charge, 4 = vice professor of nursing, 5 = professor of nursing), and monthly income (1 = less than 3,000 yuan, 2 = 3,000–4,999 yuan, 3 = 5,000–6,999 yuan, 4 = 7,000–8,999 yuan, 5 = 9,000 yuan or above).

For the elderly cancer patients, we used the following control variables: gender (1 = female, 0 = male), age (1 = 60–64 years, 2 = 65–69 years, 3 = 70 years or above), education (1 = elementary school or below, 2 = junior high school, 3 = high school or above), working status (1 = working, 0 = do not work), and tumor stage (1 = UICC stage I, 2 = UICC stage II, 3 = UICC stage III, 4 = UICC stage IV).

### 3.3. Analysis procedure

The partial least squares structural equation modeling (PLS-SEM) was conducted to address the research questions. The reasons are as follows: first, the sample in this study included both nurses and patients, and each nurse was required to be matched with a patient they care for. This resulted in a small effective sample size, whereas PLS-SEM has higher inclusiveness for sample size (Ringle et al., 2012). Secondly, the sample data in this study tended to be distributed non-normally, and PLS-SEM does not force the data to obey a normal distribution and handles non-normal distributions relatively well. Finally, PLS-SEM can better test the interaction effects among continuous variables as proposed in the theoretical model (Hair et al., 2017), such as the test of moderating effects in this study.

Data analysis was mainly completed by SPSS 26.0 and SmartPLS 3.3.9 software. SPSS was used for sample descriptive statistics and Harman’s one-factor test of common method bias test. The analysis in SmartPLS 3.3.9 was conducted through two stages (Barclay et al., 1995). The analysis of the measurement model tested the reliability and validity of all latent construct measurements. The analysis of the structural model assessed the relationships among the latent constructs for hypothesis testing.

## 4. Results

### 4.1. Sample descriptive statistics

The demographic information of the participants is shown in Table 1. Most nurses are female (99.57%), and only one male nurse is interviewed. In terms of age, 23.48% of nurses are 30 years old or below, and 42.17% are 31–35 years old. Most nurses have a bachelor’s degree (91.74%) and are married (80.43%). Regarding tenure, 33.04% of nurses are 6–10 years and 21.74% are 11–15 years. The majority of the nurses are staff nurses (70.87%). In terms of title, 24.35% are nurse practitioners and 59.13% are nurse-in-charge. 83.48% of nurses have a monthly income of 7,000 yuan or above. Among the patient samples, 68.26% of patients are female and 31.74% are male. Regarding age, 57.39% of patients are 60–64 years old and 37.39% are 65–69 years old. Most patients have elementary school education or below (81.30%), get married (97.83%), and are not currently involved in work (88.26%). In terms of tumor stage, 35.65% of patients are diagnosed with UICC stage II and 26.52% are diagnosed with UICC stage III.

**Table 1:** The demographic information of the participants.

Characteristic	Group	N	Percent (%)
*Nurse*			
Gender	Female	229	99.57
	Male	1	0.43
Age	30 years or below	54	23.48
	31–35 years	97	42.17
	36–40 years	37	16.09
	41–45 years	20	8.70
	45 years above	22	9.57
Education	Technical secondary school	0	0.00
	Junior college	6	2.61
	Bachelor’s degree	211	91.74
	Master’s degree or above	13	5.65
Marital status	Married	185	80.43
	Unmarried	45	19.57
Tenure	5 years or below	38	16.52
	6–10 years	76	33.04
	11–15 years	50	21.74
	16–20 years	33	14.35
	20 years above	33	14.35
Hire type	Staff nurse	163	70.87
	Employment nurse	23	10.00
	Contract nurse	44	19.13
Title	Nurse	17	7.39
	Nurse practitioner	56	24.35
	Nurse-in-charge	136	59.13
	Vice professor of nursing	21	9.13
	Professor of nursing	0	0.00
Income	3,000 yuan below	0	0.00
	3,000–4,999 yuan	1	0.43
	5,000–6,999 yuan	37	16.09
	7,000–8,999 yuan	84	36.52
	9,000 yuan or above	108	46.96
*Patient*			
Gender	female	157	68.26
	male	73	31.74
Age	60–64 years	132	57.39
	65–69 years	86	37.39
	70 years or above	12	5.22
Education	Elementary school or below	187	81.30
	Junior high school	31	13.48
	High school or above	12	5.22
Marital status	Married	225	97.83
	Unmarried	5	2.17
Working state	Working	27	11.74
	Do not work	203	88.26
Tumor stage	UICC stage I	47	20.43
	UICC stage II	82	35.65
	UICC stage III	61	26.52
	UICC stage IV	40	17.39

### 4.2. Common method bias test

Since the three key variables of psychological capital, work engagement, and job resources were self-reported by nurses, the relationships between variables were inevitably subject to common method bias, and statistical tests for common method bias needed to be conducted. In Harman’s one-factor test (Podsakoff et al., 2003), all items of psychological capital, work engagement, and job resources of nurses were subjected to the unrotated principal component analysis. It was found that the first principal component explained only 35.879% of the total variance, which did not exceed the recommended value of 40%.

In addition, a common method factor was further added to the PLS environment for testing based on previous studies. According to the standardization process of Liang et al. (2007), a common method factor containing all latent variable indicators was added to the model. The presence of severe common method bias can be determined by comparing the variance of the actual factor loadings and the method factor loadings for each item. The results in Table 2 showed that the mean–variance of the actual factor loadings of the measurement items was 0.651, which was much larger than the mean–variance of the method factor loadings of 0.014. In summary, the common method bias is not a severe problem in this study.

**Table 2:** The result of common method bias test.

Construct	Items	R1	R1^2^	R2	R2^2^
Nurses’ psychological capital	NPC1	0.847	0.717	0.048	0.002
	NPC2	0.800	0.640	−0.068	0.005
	NPC3	0.821	0.674	0.137	0.019
	NPC4	0.785	0.616	−0.001	0.000
	NPC5	0.726	0.527	0.034	0.001
	NPC6	0.821	0.674	−0.051	0.003
	NPC7	0.717	0.514	0.017	0.000
	NPC8	0.797	0.635	−0.118	0.014
Nurses’ work engagement	NWE1	0.805	0.648	0.102	0.010
	NWE2	0.878	0.771	−0.178	0.032
	NWE3	0.888	0.788	−0.133	0.018
	NWE4	0.806	0.650	0.093	0.009
	NWE5	0.893	0.797	−0.173	0.030
	NWE6	0.694	0.481	0.285	0.081
	NWE7	0.890	0.792	−0.193	0.037
	NWE8	0.702	0.493	0.246	0.061
	NWE9	0.815	0.665	0.082	0.007
Nurses’ job resources	NJR1	0.777	0.604	0.018	0.000
	NJR2	0.802	0.643	−0.077	0.006
	NJR3	0.766	0.587	0.060	0.004
	NJR4	0.778	0.605	−0.056	0.003
	NJR5	0.792	0.628	0.081	0.007
	NJR6	0.779	0.607	0.073	0.005
	NJR7	0.829	0.686	−0.009	0.000
	NJR8	0.867	0.751	−0.032	0.001
	NJR9	0.861	0.741	−0.047	0.002
AVG	AVG	0.805	0.651	0.005	0.014

### 4.3. Measurement model analysis

The validity of the measurement model was evaluated by testing the reliability and validity of the measurement instrument. As shown in Table 3, in terms of reliability, Cronbach’s alpha coefficient and the composite reliability of each construct are above 0.9, which exceeds the 0.70 threshold, thus confirming the reliability of the measurement instrument. Regarding validity, convergent validity is examined by the factor loadings for each item and the average variance extracted (AVE) of the constructs. The results in Table 3 suggest that the factor loadings (range 0.717–0.930) of each item reach the 0.70 threshold (Hair et al., 2019), and the AVE of each construct is above 0.6, exceeding the acceptable threshold value of 0.50 (Fornell and Larcker, 1981). It indicates that the measurement instrument has sufficient convergent validity. Finally, the discriminant validity is tested based on the Fornell-Larcker criterion and the heterotrait-monotrait (HTMT) ratio of correlations. As given in Table 4, the correlation coefficients between the constructs are less than the square root of the AVE (the value on the diagonal). According to the Fornell-Larcker criterion (Fornell and Larcker, 1981), there is good discriminant validity between the constructs. Furthermore, in Table 5, the heterotrait-monotrait (HTMT) ratio of the correlation are below the 0.90 threshold (Hair et al., 2017), which also verifies the discriminant validity. In summary, the reliability and validity of the measurement instrument are satisfactory and can be used for subsequent structural model analysis.

**Table 3:** The reliability and convergent validity of the constructs.

Construct	Items	Loadings	AVE	Composite reliability	Cronbach’s alpha
Nurses’ psychological capital	NPC1	0.851	0.624	0.930	0.914
	NPC2	0.797			
	NPC3	0.829			
	NPC4	0.783			
	NPC5	0.725			
	NPC6	0.814			
	NPC7	0.717			
	NPC8	0.797			
Nurses’ work engagement	NWE1	0.817	0.675	0.949	0.939
	NWE2	0.857			
	NWE3	0.867			
	NWE4	0.819			
	NWE5	0.872			
	NWE6	0.720			
	NWE7	0.868			
	NWE8	0.728			
	NWE9	0.827			
Nurses’ job resources	NJR1	0.839	0.645	0.942	0.932
	NJR2	0.733			
	NJR3	0.836			
	NJR4	0.718			
	NJR5	0.861			
	NJR6	0.848			
	NJR7	0.769			
	NJR8	0.802			
	NJR9	0.800			
Patient satisfaction	PS1	0.925	0.769	0.943	0.924
	PS2	0.868			
	PS3	0.789			
	PS4	0.865			
	PS5	0.930			

**Table 4:** Discriminant validity of the constructs—Fornell-Larcker criterion.

Construct	NPC	NWE	NJR	PS
NPC	**0.790**			
NWE	0.481	**0.821**		
NJR	0.249	0.254	**0.803**	
PS	0.664	0.641	0.202	**0.877**

The off-diagonal values are the correlations between the latent constructs and the diagonal values (indicated in bold) are the square values of the AVE.

**Table 5:** Discriminant validity of the constructs—HTMT criterion.

Construct	NPC	NWE	NJR	PS
NPC				
NWE	0.517			
NJR	0.265	0.261		
PS	0.716	0.683	0.211	

### 4.4. Structural model analysis

**Figure 2:**
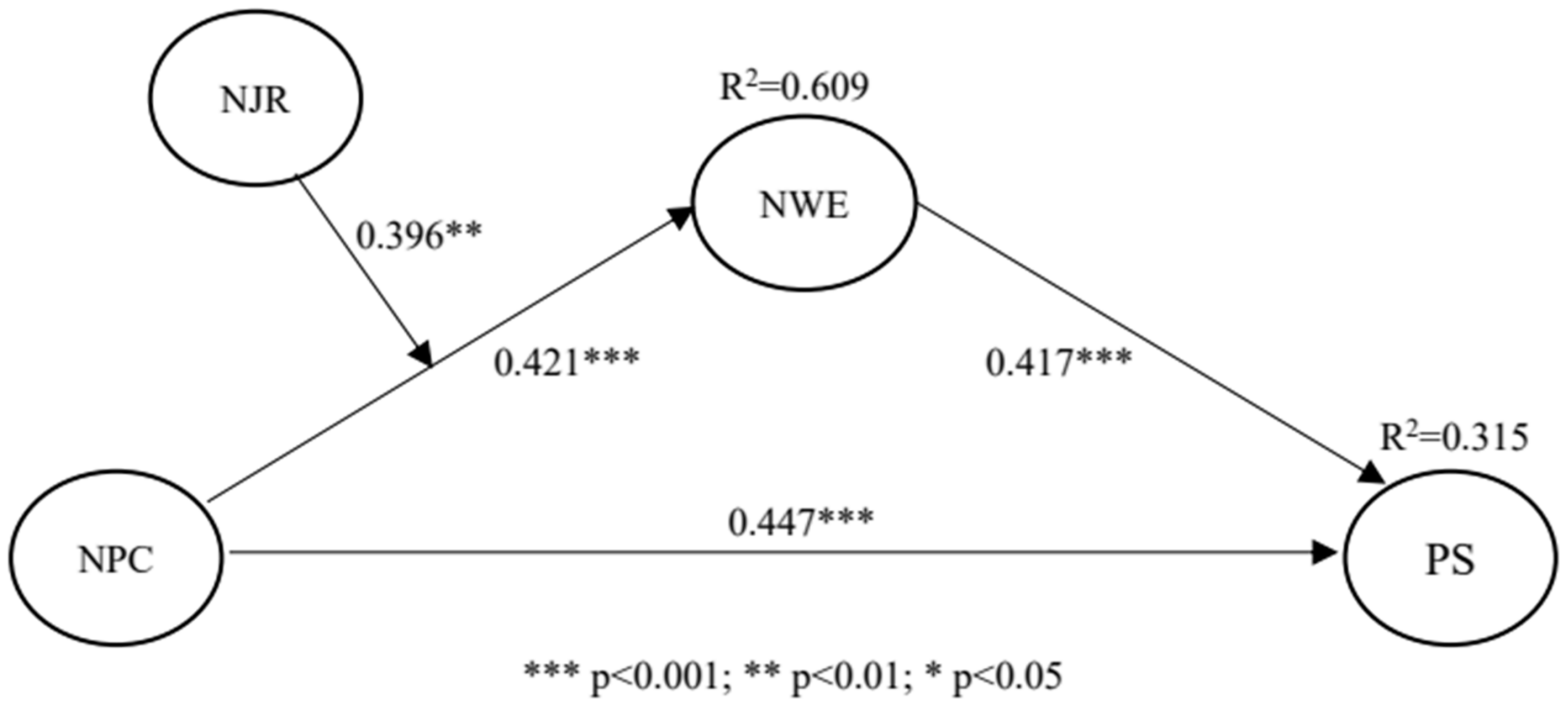
Path coefficients and *R*^2^ of the structural model.

In the evaluation of the structural model, the bootstrap method based on deviation correction was used to calculate standard errors and significance (Hair et al., 2017). The sample size was set to 5,000, and 95% CI was obtained. The path coefficient test of the structural model is shown in Figure 2 and Table 6. The results indicate that nurses’ psychological capital has a significant positive effect on patient satisfaction (*β* = 0.447, t = 8.629, *p* < 0.05) and their work engagement (*β* = 0.421, *t* = 7.061, *p* < 0.05). Therefore, H1 and H2 are supported. In addition, the work engagement of nurses positively influences patient satisfaction (*β* = 0.417, *t* = 7.954, *p* < 0.05), and H3 is supported.

**Table 6:** The mediating effect test results.

Hypothesis	Path	*β*	Std. Error	*t*	*p*	Result
H1	NPC → PS	0.447	0.052	8.629	0.000	Supported
H2	NPC → NWE	0.421	0.060	7.061	0.000	Supported
H3	NWE → PS	0.417	0.052	7.954	0.000	Supported
Test of mediation effect						
Hypothesis	Path	IE	Sobel test	TE	VAF	Result
H4	NPC → NWE → PS	0.175	5.281	0.622	28.203%	Supported

The Sobel test and variance accounted for (VAF) index were employed to examine the mediating effect (Sobel, 1982). As shown in Table 6, the Sobel test indicates that nurses’ work engagement plays a mediating role between nurses’ psychological capital and patient satisfaction (*Z* = 5.281, *p* < 0.05). The strength of the indirect effect relative to the total effect is determined by the variance accounted for (VAF) index. The criteria for mediating effect are as follows: VAF > 80% is complete mediation, 20% ≤ VAF ≤ 80% is partial mediation, and VAF < 20% has no mediation (Hair et al., 2021). The VAF for nurses’ work engagement is 28.203%. Therefore, nurses’ work engagement plays a partial mediation role between their psychological capital and patient satisfaction, and H4 is supported.

To test the moderating effect, nurses’ psychological capital was set as the predictor variable and job resources as the moderating variable in the PLS-SEM, and the interaction term was constructed. The significance of the interaction term is used as the criterion to determine the existence of moderating effect. As shown in Table 7, the interaction of nurses’ psychological capital and job resources (*β* = 0.396, *t* = 3.008, *p* < 0.05) has a significant positive effect on work engagement. It implies that nurses’ job resources play a positive moderating role in the relationship between psychological capital and the work engagement of nurses.

**Table 7:** The moderating effect test result.

Path	*β*	Std. Error	*t*	*p*
NPC → NWE	0.421	0.060	7.061	0.000
NJR → NWE	0.119	0.064	1.857	0.063
NPC*NJR → NWE	0.396	0.132	3.008	0.003

**Figure 3:**
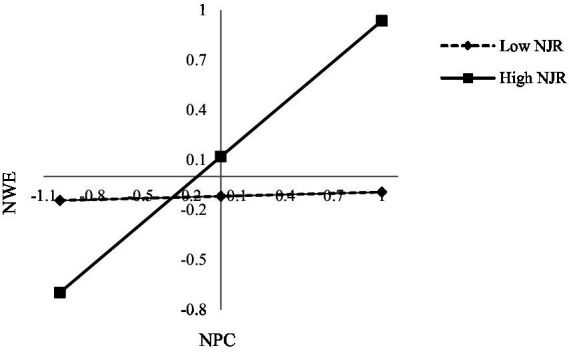
The moderating role of nurses’ job resources.

To reveal the moderating effect of nurses’ job resources more clearly, the moderating effect diagram is plotted (Figure 3). The simple slope test is conducted for the moderating effect of nurses’ job resources. The analysis results show that for nurses with high job resources, their psychological capital has a significant positive effect on work engagement (*β* = 0.817, *p* < 0.05). For nurses with low job resources, their psychological capital has a relatively small effect on work engagement (*β* = 0.025, *p* < 0.05). Thus, H5 is supported.

The measurement model was evaluated by calculating *R*^2^, effect sizes (*f*^2^), and *Q*^2^. As shown in Table 8, *R*^2^ of the endogenous constructs are examined to assess the explanatory power of the model. The *R*^2^ of nurses’ work engagement and patient satisfaction are 0.609 and 0.315, respectively, thus, the model is moderately strong (Chin, 1998). The f^2^ is used to evaluate the effect size of each predictor construct, which according to Cohen (2013) f^2^ values of 0.35, 0.15, and 0.02 are considered large, medium, and small. The effect sizes of nurses’ psychological capital and job resources on work engagement are 0.247 and 0.020. It indicates that the predictive contribution of nurses’ psychological capital to work engagement is at a medium level, while the predictive effect size of job resources on work engagement is small. The effect sizes of nurses’ psychological capital and work engagement on patient satisfaction are 0.400 and 0.345, which indicates a large predictive contribution of nurses’ psychological capital to patient satisfaction and a medium predictive contribution of nurses’ work engagement to patient satisfaction. To further assess the predictive capability of the model, *Q*^2^ values are calculated. The *Q*^2^ for the endogenous constructs nurses’ work engagement and patient satisfaction are 0.217 and 0.468. Because both of the values are greater than 0, it indicates that the model has an acceptable predictive relevance (Peng and Lai, 2012).

**Table 8:** The evaluation of measurement model fit.

Constructs	*R* ^2^	*Q* ^2^	*f*^2^ in relation to	
			NWE	PS
NPC	–	–	0.247	0.400
NJR	–	–	0.020	–
NWE	0.609	0.217	–	0.345
PS	0.315	0.468	–	–

## 5. Discussion

During the COVID-19 pandemic, as a special vulnerable group, elderly cancer patients are faced with multiple pressures such as prolonged tumor treatment and COVID-19 infection, and they are more prone to negative emotions. Therefore, the mental health of elderly cancer patients has attracted much attention. The nurses are the group with frequent contact with patients and have an important impact on patients. This prompts the researchers to explore the antecedents of patient satisfaction from the perspective of the medical staff. In the process of sorting out the influencing factors of patient satisfaction, we have found that the psychological capital of nurses is an important individual-level factor, and the research on how it affects patient satisfaction is still insufficient. Based on the JD-R model, this study explores the impact of nurses’ psychological capital on the satisfaction of elderly cancer patients in the context of the COVID-19 pandemic, with nurses’ work engagement as the mediating variable and their job resources as the moderating variable. The research conclusions are as follows.

Firstly, the psychological capital of nurses has a positive impact on the satisfaction of elderly cancer patients. Elderly cancer patients cared for by nurses with high psychological capital will experience higher satisfaction. This finding is consistent with previous studies on psychological capital as a major factor behind the promotion of job performance (Sun et al., 2012). Even under the COVID-19 pandemic, as an important strategic resource, the psychological capital of nurses can still have a positive impact on their work performance, such as the satisfaction of elderly cancer patients. Nurses with more positive psychological capital can reasonably infer their ability and have a strong ability to resist pressure. They often are optimistic about the future, know how to alleviate work pressure, and can digest bad emotions in time. Nurses convey positive emotions to patients (Brunetto et al., 2016), which can relieve anxiety, depression, and other negative emotions of elderly cancer patients. It is beneficial for patients to build up the confidence to overcome the disease and promote patients to actively accept treatment. This helps to improve the quality of care and job performance, and ultimately significantly increases the satisfaction of elderly cancer patients. The present study enriches the antecedents of satisfaction of elderly cancer patients from the perspective of nurses’ psychological capital.

Secondly, work engagement plays a mediating role between the psychological capital of nurses and elderly cancer patients’ satisfaction. When nurses have high psychological capital and they are in a positive psychological state, they are more able to perceive the significance of nursing work and have a sense of responsibility for work. It makes nurses more internal motivation to actively participate in the work and has a strong work engagement to improve the quality of care (Pahlevan Sharif et al., 2018). During the COVID-19 pandemic, elderly cancer patients are more psychologically vulnerable and feel more anxious than other groups, they eager to receive more care from nursing staff. When nurses have a high degree of work engagement, they are more likely to adopt good work behavior to provide high-quality nursing services for elderly cancer patients, which can meet the various needs of patients and bring high patient satisfaction. The present study provides empirical evidence for the mediating role of nurses’ work engagement in psychological capital and satisfaction of elderly cancer patients.

Thirdly, job resources positively moderate the relationship between psychological capital and the work engagement of nurses. Compared with the lack of job resources, the positive relationship between nurses’ psychological capital and their work engagement is stronger under rich job resources. This finding is consistent with previous research on job resources, such as support from colleagues and supervisors, which is crucial to improving the work engagement of employees (Hussein and Amiruddin, 2020). Organizational support has a positive moderating effect on the relationship between psychological capital and work engagement (Rara, 2019). Under the special background of the COVID-19 pandemic, the job responsibilities of nurses caring for elderly cancer patients include not only providing high-quality care for patients but also cooperating with epidemic prevention and control. Nurses face greater uncertainty about the external environment and work pressure, and they are more likely to produce negative emotions, which will lead to job burnout and reduce work engagement. In this case, when nurses are supported by abundant job resources, they can effectively alleviate the negative impact of the epidemic. Besides, the internal resource of nurses’ psychological capital can be deeply stimulated, and the positive impact of psychological capital on work engagement can be strengthened. In this study, nurses’ job resources are selected as the moderating variable at the organizational level to explore its moderating effect on the relationship between psychological capital and work engagement of nurses, deepening the research on the boundary effect between them.

### 5.1. Practice implications

Patient satisfaction is an important performance indicator for nurses, and measuring satisfaction levels also provides the basis for improving healthcare systems (Chung et al., 2020). This study explores the mechanism of nurses’ psychological capital on the satisfaction of elderly cancer patients. It provides practical implications for healthcare organizations to carry out effective medical staff management and obtain competitive advantages.

Firstly, healthcare organizations should realize that when nurses have positive psychological capital, elderly cancer patients will receive higher-quality medical services. Therefore, the psychological capital of nurses can be an important means to enhance the competitive advantage of healthcare organizations. As psychological capital is learnable, it can be measured, developed, and managed effectively, and it is relatively lasting and stable after being formed. Through targeted training, healthcare organizations can develop and manage the psychological capital of nurses to obtain high patient satisfaction and ultimately enhance their competitive advantage. The specific measures include: first, the physical and mental health of nurses should be put in the first place in healthcare organization management to ensure the development of positive psychological capital. Second, cultivate nurses’ positive expectations of job performance, career development and role orientation by formulating reasonable career development goals, and career planning. Third, healthcare organizations should fully endow nurses with work autonomy to cultivate their professional self-confidence and optimism. Fourth, it is necessary to build a learning and development platform for nurses to improve their self-efficacy.

Secondly, healthcare organizations can encourage nurses to make reasonable use of psychological capital to improve work engagement, to achieve good work performance. Healthcare organizations should fully investigate and understand the psychological capital of nurses and take personalized intervention measures to help nurses develop positive psychological capital. Intervention measures can be provided through traditional classroom training, video discussion, self-centered exercise, and reading intervention. By learning how to enhance self-efficacy, hope, optimism, and resilience, nurses can more effectively protect, increase, and maintain psychological capital, which translates into a series of positive outcomes such as engagement. In addition to training, healthcare organizations should take psychological capital as an important assessment indicator when recruiting medical staff. It should select and hire employees with high psychological capital characteristics so that they can quickly put into work and achieve high performance.

Finally, healthcare organizations should be aware of the importance of providing sufficient work resources for nurses, it can maximize the role of nurses’ resources and promote them to produce high work engagement. Nurses have a huge workload and high job demands. It is difficult for nurses to efficiently complete work objectives only by relying on their resources. Healthcare organizations need to provide supporting job resources such as work autonomy, career development opportunities, and social support to help nurses perform their duties. With the effective supply of these job resources, the personal psychological capital of nurses can be strengthened, so that nurses can respond to high-intensity nursing work with a positive attitude. It can also help nurses alleviate the negative impact of work pressure and be competent for higher job demands. Healthcare organizations can use job resources to effectively stimulate the work enthusiasm, initiative, and potential of nurses, which will improve their work engagement and achieve personal and organizational goals.

### 5.2. Limitations and future research

There are three shortcomings in this study that can be addressed in future studies. Firstly, we collected data on nurses’ psychological capital, job resources, work engagement, and patient satisfaction by questionnaire at the same time point. Although this method has good external validity, the cross-sectional study design has relatively insufficient internal validity and does not allow us to explore whether the relationship between nurses’ psychological capital and the satisfaction of elderly cancer patients has changed over time. Therefore, to further confirm the relationship between the variables revealed in this study, future studies can incorporate experimental methods and longitudinal follow-up surveys to enhance the validity of the study and the findings. Secondly, for sampling convenience, the sample of this study was sourced from a tertiary first-class hospital in China. Although the findings were consistent with theoretical predictions, the generalizability of the findings was limited and the applicability of the results of this study to other countries needs to be further tested. Therefore, future studies can be conducted in different countries or cultural contexts to verify the generalizability of the study. Finally, this study focused on the impact of nurses’ psychological capital on patient satisfaction at the individual level. Some scholars recently have extended psychological capital to the collective and team level, they borrowed the concept of an agency vehicle and found that psychological capital at the collective level can act as a mediator between team behavior and team performance. In conjunction with current trends in multi-level and cross-level research, future research can examine the impact of the collective psychological capital of nurses on patient satisfaction.

## Data availability statement

Questionnaire survey datasets were analyzed in this study. The raw data supporting the conclusions of this article will be made available by the authors, without undue reservation.

## Ethics statement

Ethical approval was given by the Ethics Review Board of University (EC-2021-211). An informed consent form was signed by all participants before participating in the research.

## Author contributions

HH and NZ designed the study and wrote the main part of the manuscript. NZ, BL, and SZ organized the database and performed the statistical analysis. BL and SZ are corresponding authors and supervise the project. All authors contributed to the article and approved the submitted version.

## Funding

This research was supported by the National Social Science Foundation of China (Award no. 20BGL211).

## Conflict of interest

The authors declare that the research was conducted in the absence of any commercial or financial relationships that could be construed as a potential conflict of interest.

## Publisher’s note

All claims expressed in this article are solely those of the authors and do not necessarily represent those of their affiliated organizations, or those of the publisher, the editors and the reviewers. Any product that may be evaluated in this article, or claim that may be made by its manufacturer, is not guaranteed or endorsed by the publisher.
